# Characterization of an expanded set of assays for immunomodulatory proteins using targeted mass spectrometry

**DOI:** 10.1038/s41597-024-03467-x

**Published:** 2024-06-25

**Authors:** Jeffrey R. Whiteaker, Lei Zhao, Regine M. Schoenherr, Dongqing Huang, Jacob J. Kennedy, Richard G. Ivey, Chenwei Lin, Travis D. Lorentzen, Simona Colantonio, Tessa W. Caceres, Rhonda R. Roberts, Joseph G. Knotts, Joshua J. Reading, Candice D. Perry, Sandra S. Garcia-Buntley, William Bocik, Stephen M. Hewitt, Amanda G. Paulovich

**Affiliations:** 1 Translational Science and Therapeutics Division, Fred Hutchinson Cancer Center, Seattle, WA USA; 2 Cancer Research Technology Program, Antibody Characterization Lab, Frederick National Laboratory for Cancer Research, Frederick, MD USA; 3 Experimental Pathology Laboratory, Laboratory of Pathology, Center for Cancer Research, National Cancer Institute, National Institute of Health, Bethesda, MD USA

**Keywords:** Tumour immunology, Tumour biomarkers, Medical and clinical diagnostics, Mass spectrometry

## Abstract

Immunotherapies are revolutionizing cancer care, but many patients do not achieve durable responses and immune-related adverse events are difficult to predict. Quantifying the hundreds of proteins involved in cancer immunity has the potential to provide biomarkers to monitor and predict tumor response. We previously developed robust, multiplexed quantitative assays for immunomodulatory proteins using targeted mass spectrometry, providing measurements that can be performed reproducibly and harmonized across laboratories. Here, we expand upon those efforts in presenting data from a multiplexed immuno-oncology (IO)-3 assay panel targeting 43 peptides representing 39 immune- and inflammation-related proteins. A suite of novel monoclonal antibodies was generated as assay reagents, and the fully characterized antibodies are made available as a resource to the community. The publicly available dataset contains complete characterization of the assay performance, as well as the mass spectrometer parameters and reagent information necessary for implementation of the assay. Quantification of the proteins will provide benefit to correlative studies in clinical trials, identification of new biomarkers, and improve understanding of the immune response in cancer.

## Background & Summary

Cancer immunotherapies, including checkpoint inhibitors, cancer vaccines, and adoptive cell therapies, have shown tremendous potential to induce durable responses in several cancer types^1,2^. However, response rates vary widely, with subgroups of patients benefitting and the possibility of a wide spectrum of toxicities^3,4^. Understanding the mechanisms of response and resistance to immunotherapies is necessary to develop better predictive biomarkers, match patients to optimum therapies, and predict and monitor immune related adverse events (irAEs).

There are hundreds of proteins involved in the immune response to cancer^5,6^. Quantifying those immunomodulatory proteins to better understand how treatments alter expression is a tremendous challenge. Traditional technologies, like Western blot or immunohistochemistry (IHC), rely on highly characterized, monospecific antibodies, which can be susceptible to interferences and/or cross-reactivity^7^. In addition, these approaches are typically performed one analyte at-a-time, are semi-quantitative, and are difficult to standardize across laboratories. New technologies, like proteomic sensors incorporating modified aptamers^8^ have greatly improved throughput and multiplexing capabilities, but do not directly detect the protein, relying on transducers and thus resulting in some of the same specificity challenges facing traditional immunoassays^9^. In contrast, mass spectrometry-based assays directly measure the target of interest, offering potential absolute specificity^10^. When coupled with stable isotope dilution approaches for quantification, targeted mass spectrometry-based proteomic assays offer highly multiplexed quantification that can be standardized and harmonized across laboratories^11,12^.

We previously developed two panels of multiplexed targeted mass spectrometry-based assays to quantify proteins related to the cancer immunity cycle to advance immuno-oncology (IO) efforts^13,14^. The first panel (the “IO-1” assay) quantified 46 immunomodulatory proteins^13^, whereas the second panel (the “IO-2” assay) quantified 43 proteins^14^. The panels use peptide immunoaffinity enrichment coupled with multiple reaction monitoring mass spectrometry (immuno-MRM) to enrich and analyze low abundance peptides, as stoichiometric representatives of the target proteins^15^. Quantification is performed using stable isotope-labeled peptides as internal standards, spiked into each sample at a known concentration, allowing for highly robust and reproducible analysis that can be readily multiplexed^16,17^.

Here, we extend upon the previous work by developing and characterizing forty monoclonal antibodies and incorporating them into a novel “IO-3” assay panel, significantly expanding the capabilities for the quantification of immunomodulatory proteins. The assay targets 43 peptides to 39 proteins (3 antibodies target a modified and unmodified form of the same peptide) and can be used in conjunction with the previously developed IO-1 and IO-2 assays. The dataset described herein presents all aspects of assay development and characterization, including evaluation of the performance of the monoclonal antibodies used in the assay, parameters for multiple reaction monitoring of the targeted peptides, fit-for-purpose bioanalytical validation of the multiplexed panel in tissue and plasma matrices, and characterization of the expected performance of the assays in a panel of biospecimens.

## Methods

### Reagents

Light (unlabeled) synthetic peptides were obtained from Biosynth (Gardner, MA, USA) as crude (flash purified) grade. Cleavable stable isotope-labeled (heavy) peptides (i.e., containing additional amino acids on the ends of tryptic cut sites) were obtained from Biosynth and were purified >95% by HPLC, labeled with [^13^C and ^15^N] at the tryptic C-terminal Arg or Lys, and quantified by amino acid analysis (AAA). Aliquots of peptide standards were stored in 5% acetonitrile/0.1% formic acid at −80 °C until use. Rabbit monoclonal antibodies (mAbs) were produced with Epitomics/Abcam (Cambridge, MA, USA) and Excel Biopharm (Burlingame, CA, USA). Mouse monoclonal antibodies were produced with Precision Antibody (Columbia, MD, USA) and the Antibody Development Facility at the Fred Hutchinson Cancer Center (Seattle, WA, USA). Urea (#U0631), Trizma base (#T2694), citric acid (#C0706), dimethyl sulfoxide (DMSO, #D2438), EDTA (#E7889), EGTA (#E0396), and iodoacetamide (IAM, #A3221) were obtained from Sigma (St. Louis, MO, USA). Acetonitrile (MeCN, #A955), water (#W6, LCMS Optima® grade), trifluoroacetic acid (TFA, LC-MS grade, #85183), tris(2-carboxyethyl)phosphine (TCEP, #77720), phosphate buffered saline (PBS, #BP-399-20), ammonium bicarbonate (A643-500), xylene (#422685000), and (3-[(3-cholamidopropyl) dimethylammonio]-1-propanesulfonate) (CHAPS, #28300) detergent were obtained from Thermo Fisher Scientific (Waltham, MA, USA). Formic acid (#1.11670.1000) was obtained from EMD Millipore (Billerica, MA, USA). Lys-C (Wako, #129-02541), trypsin (Worthington #LS003740), and sequencing grade trypsin (#V5111, Promega, Madison, WI, USA) were used for digestion of samples.

### Human samples

Frozen tissue and plasma samples were supplied by the Clinical Proteomics Tumor Analysis Consortium (CPTAC) as anonymized samples from consenting donors collected under IRB-approved protocols. Plasma samples used for fit-for-purpose validation studies were commercially acquired from BioIVT (Westbury, NY, USA).

### Sample preparation

#### Protein extraction

To produce lysates for immuno-MRM analysis, frozen human tissue was cryopulverized in a cryoPREP CP-02 (Covaris, Woburn, MA, USA) and stored frozen until analysis. 5 μL of lysis buffer (pH 8.5; 25 mM Tris, 6 M Urea, 1 mM EDTA, 1 mM EGTA, 1% (v/v) Sigma protease inhibitor (#P8340), 1% (v/v) Sigma phosphatase inhibitor cocktail 2 (#P5726), 1% (v/v) Sigma phosphatase inhibitor cocktail 3 (#P0044)) was added for each mg wet tissue weight (up to 1000 μL). The sample was vortexed for 10–15 sec and sonicated three times in a cup horn probe (filled with ice water) at 50% power for 30 seconds. Protein concentration was determined using Micro BCA Protein Assay Kit (Pierce, #23235) prior to storage in the vapor phase of liquid nitrogen until the day of digestion.

#### Enzymatic digestion

A mix of cleavable stable isotope-labeled peptide standards was added to the lysate at 200 fmol/sample. 500 μg frozen tissue lysates was transferred to a deep-well plate for processing on an EpMotion 5057 (Eppendorf). Lysates were reduced in 30 mM TCEP for 30 minutes at 37 °C with shaking, followed by alkylation with 50 mM IAM at room temperature. Lysates were then diluted with 0.8 mL 200 mM TRIS (pH 8.0) before Lys-C endopeptidase was added at a 1:50 enzyme:substrate ratio by mass and incubated for 2 hours at 37 °C with mixing at 600 rpm (Thermomixer, EpMotion 5057). After 2 hours, trypsin was added at a 1:50 enzyme:substrate ratio. Digestion was carried out overnight at 37 °C with mixing at 600 rpm. After 16 hours, the reaction was quenched with formic acid (final concentration 1% by volume).

Human plasma (2 × 50 μL aliquots of each sample) was denatured with 150 μL of lysis buffer (25 mM Tris, 6 M Urea, 1 mM EDTA, 1 mM EGTA, 1% (v/v) Sigma protease inhibitor (#P8340), 1% (v/v) Sigma phosphatase inhibitor cocktail 2 (#P5726), 1% (v/v) Sigma phosphatase inhibitor cocktail 3 (#P0044)). The plasma was reduced with 30 mM TCEP at 37 °C for 30 min, and alkylated with 50 mM IAM at room temperature for 30 min. Urea concentration was diluted 10-fold with 200 mM Tris (pH 8.0) prior to overnight digestion at 37 °C with Lys-C/trypsin using a 1:50 (w/w) enzyme:substrate. Digestions were terminated with 1% formic acid. The digest mixture was desalted using Oasis HLB 96-well plates (Waters #WAT058951) and a positive pressure manifold (Waters #186005521) according to the following procedure: wash cartridge with 4 × 400 μL of 50% acetonitrile in 0.1% formic acid, equilibrate with 4 × 400 μL of 0.1% formic acid, load total volume of digest, wash with 4 × 400 μL of 0.1% formic acid, and elute with 3 × 400 μL of 50% acetonitrile in 0.1% formic acid. The eluates were lyophilized and stored at −80 °C.

#### Peptide immunoaffinity enrichment

Enrichment was performed as previously described^18^, with the following modifications. The final assay consisted of a mixture of 40 antibodies. Antibodies were crosslinked on protein G beads (GE Mag Sepharose, Cytiva #28-9513-79), and peptide enrichment was performed using 1 μg antibody - protein G magnetic beads for each target. Digested lysate was resuspended in 200 μL 1X PBS + 0.01% CHAPS (pH was adjusted to 7.0 with 10 μL of 1 M Tris, pH 9). For plasma, the two digestion aliquots were combined after resuspension to a total volume of 200 μL. Beads were mixed in the incubation plate, washed twice in 1X PBS buffer + 0.01% CHAPS, washed once in 1/10X PBS + 0.01% CHAPS, and peptides were eluted in 26 μL of 5% acetic acid/3% acetonitrile/50 mM citrate. The elution plate was covered with adhesive foil and frozen at −80 °C until analysis.

### LC-MRM analysis

LC-MS was performed with an Eksigent 425 nanoLC system with a nano autosampler and chipFLEX system (Eksigent Technologies, Dublin, CA) coupled to a 5500 QTRAP mass spectrometer (SCIEX, Foster City, CA). Peptides were loaded on a trap chip column (Reprosil C18-AQ, 0.5 mm × 200 μm, SCIEX, #804-00016) at 5 μL/min for 3 minutes using mobile phase A (0.1% formic acid in water). The LC gradient was delivered at 300 nL/minute and consisted of a linear gradient of mobile phase B (90% acetonitrile and 0.1% formic acid in water) developed from 2–14% B in 1 minute, 14–34% B in 20 minutes, 34–90% B in 2 minutes, and re-equilibration at 2% B on a 15 cm × 75 μm chip column (ChromXP 3C18-CL particles, 3 μm, SCIEX, #804-00001). Column temperature was maintained at 45 °C. The nano electrospray interface was operated in the positive ion MRM mode. Parameters for declustering potential (DP) and collision energy (CE) were taken from optimized values in Skyline^19,20^. Scheduled MRM transitions used a retention time window of 210 seconds and a desired cycle time of 1.5 seconds, enabling sufficient points across a peak for quantification. A minimum of two transitions per peptide, including endogenous and spiked heavy peptides, were recorded for each light and heavy peptide.

### Data analysis

MRM data were analyzed using Skyline. Peak integrations were reviewed manually, and transitions from analyte peptides were confirmed by the same retention times and relative transition areas of the light peptides and heavy stable isotope-labeled peptides. Transitions with detected interferences were not used in the data analysis. Integrated raw peak areas were exported from Skyline and total intensity was calculated using Peak Area + Background. Transitions were summed for each light/heavy pair and peak area ratios were obtained by dividing peak areas of light peptides by that of the corresponding heavy peptides (or vice versa for response curves). All measurements were filtered by the LLOQ (i.e., all measurements were required to be above the LLOQ). Peak area ratios were log (base 2) transformed for statistical analysis.

Minimum tissue requirements were calculated by using frozen tissue results of signal-to-noise levels measured at 500 μg input. The signal-to-noise levels were compared to the LLOQ for each analyte using a one-sample t-test and those analytes with 95% confidence above LLOQ were further converted to input mass using linear scale dilution. The confidence interval in the minimum tissue calculation was determined based on the standard deviation of signal-to-noise distribution and degrees of freedom (equal to the number of samples for each tissue site ‐1).

### Fit-for-purpose assay validation

The analytical performance of the assay was characterized in tissue and plasma matrices to establish figures of merit for: (i) response curves, (ii) repeatability, (iii) stability, and (iv) sequential enrichment of multiple assays from a single sample aliquot.

#### Response curve

Background matrices consisting of an equal mixture of protein lysate from 4 frozen tumors or commercially obtained plasma were used to generate response curves. Aliquots (500 μg of tissue, 100 μL plasma) of the matrix were spiked with cleavable heavy stable isotope-labeled peptides at 2000, 200, 20, 8, 3.2, 1.28, 0.512, or 0.205 fmol prior to digestion. 200 fmol of light peptide was also spiked into the digested lysate. Blanks were prepared using background matrix with light peptide (no heavy spike). All points were analyzed in process triplicate. The monoclonal antibodies were coupled to magnetic beads and used to enrich the peptides. The eluates were analyzed by LC-MRM-MS. Peptide specificity was confirmed by equivalent retention times and relative transition areas of the heavy and light peptides. Curves were analyzed using Skyline by performing linear regression using log transformed data on all points above the lower limit of quantification. The Lower Limits of Quantification (LLOQs) were obtained by empirically finding the lowest point on the curve that had CV < 20% and maintained linear correlation coefficient ≥ 0.98. The Upper Limits of Quantification (ULOQs) were determined by the highest concentration point of the response curve that was maintained in the linear range of the response. For curves that maintained linearity at the highest concentration measured, the ULOQ is a minimum estimate.

#### Repeatability

Repeatability was determined using the same matrix used to generate the response curves. Heavy peptides were spiked in at three concentrations (20, 200, 2000 fmol; low, medium, high) into 500 µg aliquots of the pooled tissue lysate or 100 µL aliquots of plasma matrix. All light peptides were added into each aliquot at 200 fmol. Complete process triplicates (including digestion, peptide enrichment, and mass spectrometry) were prepared and analyzed on five independent days. In addition to evaluating spiked peptides, the repeatability of endogenous measurements was determined by spiking cleavable heavy peptide standards into 500 µg aliquots of the pooled tissue lysate and 100 µL aliquots of plasma. Measurements were made using 5 complete process replicates of endogenous peptide over 8 days (n = 40). Peptide specificity was confirmed by equivalent retention times and relative transition areas of the heavy and light peptides. Intra-assay variation was calculated as the mean %CV obtained within each day. Inter-assay variation was the %CV calculated from the mean values of each day.

#### Stability

Stability of the enriched peptides was determined by analyzing aliquots of the medium spike level sample used in repeatability studies after storage at 4 °C in the autosampler for approximately 24 hours and after 2 freeze-thaw cycles. Each condition was measured in process triplicate.

#### Sequential enrichment

A pool of protein lysates from HeLa, MCF7, and NCI-H226 cells was created using equal mass from each lysate. Peptide immunoaffinity enrichment was conducted as described above using IO-1, IO-2, and IO-3 panels with 200 fmol of cleavable heavy standards added to independent aliquots of the lysate. Each assay panel was sequentially applied to a single individual 500 µg aliquot of the pooled digest by immediately adding antibody-coupled beads to the flow-through from a previous assay enrichment. Each enrichment was performed in triplicate.

### Characterization of antibodies

#### Cell culture and lysis for antibody characterization

Cell lines HeLa (American Type Culture Collection (ATCC), #CCL-2), Jurkat (ATCC, #TIB-152), A549 (ATCC, #CCL-185), MCF7 (ATCC, #HTB-22), and NCI-H226 (ATCC, #CRL-5826) were cultured and harvested according to vendor’s specifications. Briefly, cells were sub-cultured and after trypsinization, were centrifuged at 1500 rpm for 6 minutes and supernatant was removed and discarded. Cells were washed 1X with 1X PBS, centrifuged as before, resuspended in 1X PBS, and counted. Cells were subdivided according to desired cell number in 15 mL centrifuge tubes and centrifuged as before. Cell pellets were frozen and stored at −80 °C until whole cell lysis using RIPA lysis and extraction buffer (Thermo Fisher Scientific, #89900) following the manufacturer’s protocol. Halt Protease & Phosphatase Single-Use Inhibitor Cocktail (Thermo Fisher Scientific, #78442) and Pierce Universal Nuclease for Cell Lysis (Thermo Fisher Scientific, #88701) were added according to manufacturer’s instructions. Lysate protein concentration was estimated with a BCA assay (Pierce, #23225), as described by the manufacturer’s instructions. Buffy coat (Rockland Antibodies and Assays, #R614-0100) and PBMC (ATCC, #PCS-800-011) were lysed by adding M-PER lysis buffer. The cells were lysed by pipetting the crude cell lysate 5X on ice.

#### Tissue Lysis for antibody characterization

Tissue samples for immunoblot and simple western analysis were acquired from Bio-IVT (Westbury, NY) and processed using CP02 Automated Dry Pulverizer (Covaris), following the vendor’s protocol. Briefly, tissue samples of approximately 1 g were chilled in liquid nitrogen for about 30 seconds, then mechanically pulverized with three impacts (impact level 6). Pulverized tissues were lysed using the same procedure described above for cell lysates.

#### Immunoblot

Recombinant proteins and over-expressed cell line lysates were obtained from Origene (Rockville, MD, USA) or Novus Biologicals (Littleton, CO, USA). Western blots were performed according to SOP#M-103.v1, published at the CPTAC Antibody Portal (antibodies.cancer.gov). Briefly, proper sample loading on the 4–20% Criterion TGX Stain-free precast gels (Bio-Rad, # 5678094) was verified by rapid florescence detection with ChemiDoc MP imager. Traditional immunoblotting used 10 μg/mL recombinant protein in reducing conditions (20 μL, 200 ng total protein/lane). Whole cell lysates were diluted to 2.5 mg/mL in reducing conditions (20 μL, 50 μg total protein/lane). Transfers of protein from precast gels were performed by Bio-Rad Turbo-Blot at “High MW” setting for 10 minutes. Blocking of the membrane was performed using Bio-Rad Blotting Grade Blocker (Bio-Rad, #1706404) at 5% in 1X PBS/0.5% Tween-20. Primary antibodies (1 mg/mL) were diluted in 1X PBS/0.5% Tween-20 to a dilution of 1:5000 at a total volume of 25 mL. Washing of membrane was conducted using 1X PBS/0.5% Tween-20 three times. Secondary HRP-linked rabbit specific antibody (#111-035-144, Jackson ImmunoResearch, West Grove, PA, USA) or secondary HRP-linked mouse antibody (#115-035-062, Jackson Immunoresearch Laboratories) was diluted at 1:5000 in 1 X PBS/0.5% Tween-20 at a final volume of 25 mL. Immuno-detection was performed using colorimetric substrate Opti-4-CN (Bio-Rad, #1708235) at 1 mL per blot or using enhanced chemiluminescence using Clarity Western ECL Substrate (BioRad, #1705061) at 1 mL per blot. Development of immunoblot was captured using Bio-Rad ChemiDoc MP imaging system.

#### Simple western

The Simple Western (Jess, ProteinSimple, USA, Cat. #004-650) system was used to detect primary antibody binding to a target protein in cell lysates (HeLa, Jurkat, A549, MCF7, NCI-H226, buffy coat, and/or PBMC) and tissue lysates (normal spleen and endometrium, tumor breast, lung, and ovary). The Simple Westerns were performed following the procedures detailed in SOP#M-134, published at the CPTAC Antibody Portal (antibodies.cancer.gov). In brief, cell lysates were run using the 12–230 kDa separation module, 8 × 25 capillary cartridges (ProteinSimple, #SM-W004) and detected with Anti-Rabbit Detection Module (ProteinSimple, #DM-001) or Anti-Mouse Detection Module (Proteinsimple, #DM-002). Cell lysates were run at a concentration of 200 μg/mL and incubated with primary antibodies diluted to 2 μg/mL.

#### Reverse phase protein array

Protein array analyses were performed using NCI-60 cell lines obtained from the Cancer Research Technology Program at NCI-Frederick (Frederick, MD, USA). The NCI-60 cell lines were collected at the log phase growth and protein prepared by resuspending cell pellets in RIPA (Thermo Fisher Scientific, #89900) per the manufacturer’s recommendations; total protein concentration was measured by BCA Protein Assay kit (Thermo Fisher Scientific, #23225). Quantification of protein expression values was performed by well-based reverse phase protein array (RPPA) as previously reported^21,22^. Briefly, five microliters (100 ng/well) of NCI-60 cell line antigens in PBST (1X PBS, 0.1% Tween-20) were applied onto 96-well Multi-Array™ plates (96 HB SECTOR Plate, Meso Scale Discovery, Gaithersburg, MD, USA). The plates were allowed to dry at room temperature for 2 hours. Prior to primary antibody incubation, the antigen-coated plates were blocked with 5% non-fat dry milk in PBST for 1 hour at room temperature. Target-specific antibodies were diluted (1:1000 to 1:5000) with 5% BSA in PBST. For each cell line, 25 μL of antibody were added and incubated overnight at 4 °C. The plates were washed with PBST and followed by a 90 min incubation with goat anti-mouse or anti-rabbit SULFO-TAG™ antibodies (Meso Scale Discovery) at a dilution of 1:2000 (0.5 µg/mL) containing 5% non-fat dry milk in PBST. Plates were washed, and MSD-T read buffer was added to the plate to detect binding signals using MESO QuickPlex SQ 120 reader (Meso Scale Discovery). PBST-coated wells were included on each plate as a control of non-specific binding. For each mAb, the electrochemical luminescence value of each cell line was normalized by the average value of the 60 cell lines. After normalization, levels below 0.5 were interpreted as weak or negative expression and levels above 1.5 were interpreted as strong, positive expression.

#### Immunofluorescence

The Standard Immunostaining Protocol, SOP#M-137.v2, located on the CPTAC Antibody Portal (antibodies.cancer.gov) was used for immunofluorescence (IF). Briefly, before starting, adherent cells (HeLa, A549, MCF7, and NCI-H226) were cultured overnight at 20,000 cells/well in 100 µL cell culture media in a 12.5 µg/mL superfibronectin (Sigma) coated 96-well glass bottom microplate (Sigma). Suspension cells (Jurkat) were resuspended at 10 million cells/mL in 1X PBS and used the Shi-fix™ coated 96-well microplate (Everest Biotech) at a recommended concentration between 200,000–500,000 cells/well. Shi-fix™ manufacturer’s instructions were followed and proceeded to fixation. The following day, cell culture media was removed from adherent cells and cells were fixed with 4% paraformaldehyde (VWR)/10% Fetal Bovine Serum (FBS, Thermo Fisher Scientific)/1 X PBS, washed with 1 X PBS, permeabilized with 0.1% Triton X-100 (Sigma)/1X PBS, blocked with 10% FBS constituted in 0.1% Triton X-100/1X PBS and incubated with diluted primary antibodies (1:150 dilution) with 1 µg/mL mouse or rabbit anti-alpha tubulin (Abcam) in blocking buffer overnight at 4 °C. The next day, primary antibodies were removed from cells and cells were washed with 1X PBS, incubated with diluted secondary antibodies (1:300 dilution anti-rabbit or anti-mouse Alexa Fluor 488 (Thermo Fisher Scientific) with 1:300 dilution anti-mouse or anti-rabbit Alexa Fluor 555 (Thermo Fisher Scientific)) and 0.2 µg/mL DAPI (VWR) in blocking buffer for 1 hour at room temperature in darkness. With minimum light present, secondary antibodies were removed from cells and cells were washed with 1X PBS and wells were filled with glycerol (Thermo Fisher Scientific)/10X PBS (Fisher Scientific). The 96 well microplate was sealed with an adhesive aluminum PCR plate seal (VWR) and stored at 4 °C until IF analysis (up to two weeks) using an Invitrogen™ EVOS™ M 5000 Imaging System (Thermo Fisher Scientific).

#### Immunoprecipitation

Antibodies were immobilized on NHS activated beads (Thermo Scientific, #88827) according to manufacturer’s instructions. Briefly, NHS activated beads were washed with ice cold 10 mM HCl then combined with antibodies diluted in 50 mM borate, pH 8.5 (Thermo Scientific, #28384), and allowed to incubate at 4 °C overnight with 1200 rpm shaking. Beads were washed with 0.1 M glycine pH 2.5 and DI water, then quenched with 3 M ethanolamine for 2 hours at room temperature. Quenched beads were washed with DI water and 1X PBS/0.05% CHAPS before being diluted to a final antibody concentration of 0.05 mg/mL in 1X PBS/0.05% CHAPS. Immunoprecipitation samples were prepared by combining ~15 μg of overexpressed lysate in 1X PBS/0.05% CHAPS and 5 μg of immobilized antibody on NHS activated beads. Samples were incubated overnight at 4 °C with 1200 rpm shaking. Sample processing took place on a KingFisher Flex system (Thermo Scientific, #5400620). Beads were washed 3 times with 1X PBS/0.05% CHAPS then eluted with 0.1 M Glycine, pH 2.5. Eluates were neutralized with 1 M Tris, pH 8.0. Eluates were screened using Simple Western as described above.

#### Immunohistochemistry

Tissue Microarray (TMA) consisting of breast, ovary, colon, lung, and prostate were constructed using a tissue arrayer (Pathology Devices, Westminster, MD). Tissue cores of 1.0 mm diameter were arrayed on a recipient paraffin block with a representative tumor area carefully selected from a hematoxylin and eosin (H&E) stained section of a donor block for each tumor. Immunohistochemical staining was performed according to protocol SOP#M-106 described on antibodies.cancer.gov. Briefly, TMA blocks were cut at 5 µm-thick sections, deparaffinized through xylene and rehydrated with graded alcohols to distilled water. Antigen retrieval was performed in a pressure cooker (Pascal; Dako, Carpinteria, CA) with pH 6.0 citrate buffer (Dako, #S2369) for 20 min. Endogenous peroxidase activity was blocked with 3% H_2_O_2_ for 10 min and incubated with an additional protein block (Dako, #X0909) to abate nonspecific staining. Subsequently, primary antibodies hybridization was carried out at an optimized dilution for 60 min at room temperature. Sections were labeled with Envision + detection system (Dako) for 30 min, then visualized using 3,3′-diaminobenzidine (Dako, #K3468). All sections were counterstained with hematoxylin and coverslipped after dehydration.

## Data Records

### Data record 1

The data record is available at *figshare*^23^ with filename “IO3 panel target and reagent details (xlsx)”^23^ containing a complete list of target analytes and assay reagents. The table contains gene symbols, target peptide sequences, extended peptide sequences used for heavy stable isotope-labeled internal standards, direct links to CPTAC Assay Portal IDs, direct links to CPTAC Antibody Portal IDs, and literature references supporting relevance to immuno-oncology for analytes targeted in the assay. Additional characterization data can be found on the CPTAC Assay Portal^24^ (https://assays.cancer.gov) and CPTAC Antibody Portal^25^ (https://antibodies.cancer.gov) with direct links provided in Data Record 1^23^.

### Data record 2

The data record is available at *figshare*^23^ with filename “IO3 panel MRM parameters (xlsx)”^23^ containing a list of optimized mass spectrometer parameters used for multiple reaction monitoring experiments. For each peptide targeted, the table contains precursor m/z values, precursor charge states, fragments selected for transitions, fragment m/z values, fragment charge states, and optimized collision energies. Light synthetic standards were used to select transitions (precursor/fragment ion pairs) and optimize collision energy parameters in the mass spectrometer.

### Data record 3

The data record is available at *figshare*^23^ with filename “IO3 panel assay characterization (xlsx)”^23^ containing performance figures of merit in bioanalytical fit-for-purpose validation experiments for the multiplexed assay panel using frozen tissue and plasma matrices. Additional characterization data can be found on the CPTAC Assay Portal (https://assays.cancer.gov) and CPTAC Antibody Portal (https://antibodies.cancer.gov). Response curve results describe the curve fit (correlation coefficient, R squared), upper and lower limits of quantification, and linear dynamic range. Repeatability of peptide measurement in each matrix is reported as the intra-assay (within day) and inter-assay (between day) CV at three concentrations of spiked peptide (low, medium, high). In addition, the intra-assay and inter-assay CVs are reported for peptides measured above the LLOQ at endogenous levels in the tissue and plasma matrices. The stability of the peptides in processed samples is also reported. Stability is presented by the percent difference in samples processed and stored in the autosampler for 24 hours or analyzed after 2 freeze-thaw cycles compared to immediate analysis.

### Data record 4

The data record^26^ is available at Panorama Public, a part of the ProteomeXchange consortium for dissemination of proteomics data^27^, containing processed (.sky) and raw (.wiff,.wiff.scan) mass spectrometry data files for use of multiple assay panels on a single biospecimen. The data record also contains a list of all data files and mapping for raw and processed results (found in the Supplementary Data tab on Panorama) from using the IO-3 assay in combination with previously characterized IO-1 and IO-2 immunomodulatory assay panels. The purpose was to validate a sequential enrichment approach, where the flow-through of a sample enrichment is used to enrich the target analytes from other assay panels. The data files contain results from the three IO assay panels used in the multiple enrichment approach.

### Data record 5

The data record is available at Panorama Public containing processed (.sky) and raw (.wiff,.wiff.scan) mass spectrometry data files for application of the IO-3 panel to a collection of tissue and plasma samples^26^. The data files are from measuring endogenous peptide in a panel of tissue and plasma samples. The purpose was to profile the expected detection rates for the analytes in the panel and determine sample requirements for larger studies.

### Data record 6

The data record is available at *figshare* with filename “IO3 panel antibody characterization (xlsx)”^23^ containing results from extensive characterization of the antibodies in a variety of other assay formats. All antibodies were tested in Western blotting, Simple Western, and Reverse Phase Protein Array using recombinant proteins and/or over-expressing lysates. Antibodies found positive in those tests were further screened in five applications: (i) Western blot using a panel of tissue lysates, (ii) Simple Western using a panel of tissue lysates, (iii) immunofluorescence using HeLa cells, (iv) immunoprecipitation against over-expressing cell line lysates, and (v) immunohistochemistry (IHC) against a tumor microarray.

## Technical Validation

### Method development

**Fig. 1:**
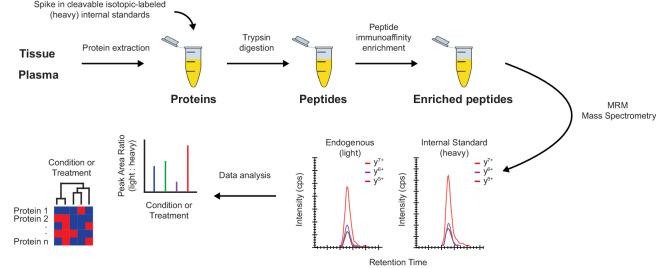
The immuno-MRM workflow allows for highly specific and sensitive multiplexed quantification of proteins. The assay workflow begins by generating a protein lysate from the biospecimen. Synthetic, cleavable stable isotope-labeled peptides for each targeted sequence are spiked into the sample at a known concentration to act as internal standards. The protein mixture is enzymatically (Lys-C and trypsin) digested to convert the lysate to a mixture of peptides. Custom monoclonal antibodies coupled to magnetic beads are used to enrich the endogenous peptides and labeled standards, and the eluate is analyzed by multiple reaction monitoring-mass spectrometry, targeting multiple transitions (i.e., combinations of precursor/fragment ion pairs). The endogenous analyte peptides and internal standards coelute (i.e., they feature equivalent retention times) and the relative areas of monitored transitions are used to confirm specificity. The amount of endogenous peptide is quantified relative to the heavy standard.

The immuno-MRM assay quantifies peptides as surrogates for the proteins of interest (see Fig. 1). Proteins for the IO-3 panel were identified as proteins involved in immunomodulatory functions from the literature (Data Record 1)^23^ and expert consultation. Proteotypic peptides to the proteins were selected by using empirical observations from existing LC-MS/MS datasets^28–40^ to generate a list of peptides most frequently detected by mass spectrometry. Peptides were ranked based on the intensity and frequency of observations in the LC-MS/MS datasets^28–40^. Guidance for peptide selection was based on previously described criteria^41,42^. Peptides were required to be unique to the protein of interest, fully tryptic (i.e., no missed cleavages), internal KP and RP sites were allowed, and neighboring trypsin cleavage sites (ragged ends) were deprioritized (i.e., selected only when other peptides were not available). Ile/Leu substitution was not considered in matching peptides to proteins, except in the case of known variants (see below). Protein N-terminal peptides were avoided, and protein C-terminal peptides were deprioritized (however, no C-terminal peptides were used for these targets). Peptide hydrophobicity, determined by SSRCalc^43^, was required to be between 10–40, and length (number of amino acids) was 5–30. For amino acid composition, C and M (oxidation) were deprioritized, NP and NG (deamidation) were avoided, and N-terminal Q (formation of pyroglutamate) was avoided. Single nucleotide variations for each protein were annotated from dbSNP (https://www.ncbi.nlm.nih.gov/snp) and peptides containing a variant with minor allele frequencies greater than 1% of the population were avoided. Finally, peptides containing post-translational modifications detected frequently in empirical datasets or reported frequently in databases^44^ were avoided. A total of 43 peptides, representing 39 proteins, were selected for assay development (Data Record 1)^23^.

To develop immuno-MRM assays for the selected peptides, we obtained three custom reagents: (i) anti-peptide monoclonal antibodies, (ii) light synthetic tryptic peptides, and (iii) cleavable stable isotope-labeled synthetic peptide standards. Monoclonal antibodies used for peptide immunoaffinity enrichment were generated according to established procedures^45^. We used 40 monoclonal anti-peptide antibodies for the IO-3 panel (some antibodies target multiple peptides). Light synthetic tryptic peptides identical in sequence to the target peptides were used to optimize parameters for multiple reaction monitoring mass spectrometry (MRM-MS), namely fragment ion selection, collision energy optimization, and retention time determination (Data Record 2)^23^. Finally, cleavable stable isotope-labeled peptides, incorporating a heavy lysine/arginine and 2–5 additional amino acids from each terminal tryptic cut site, were synthesized to use as standards that are added prior to the trypsin digestion step in order to control for variation in sample processing.

### Linearity and limits of quantification

**Fig. 2:**
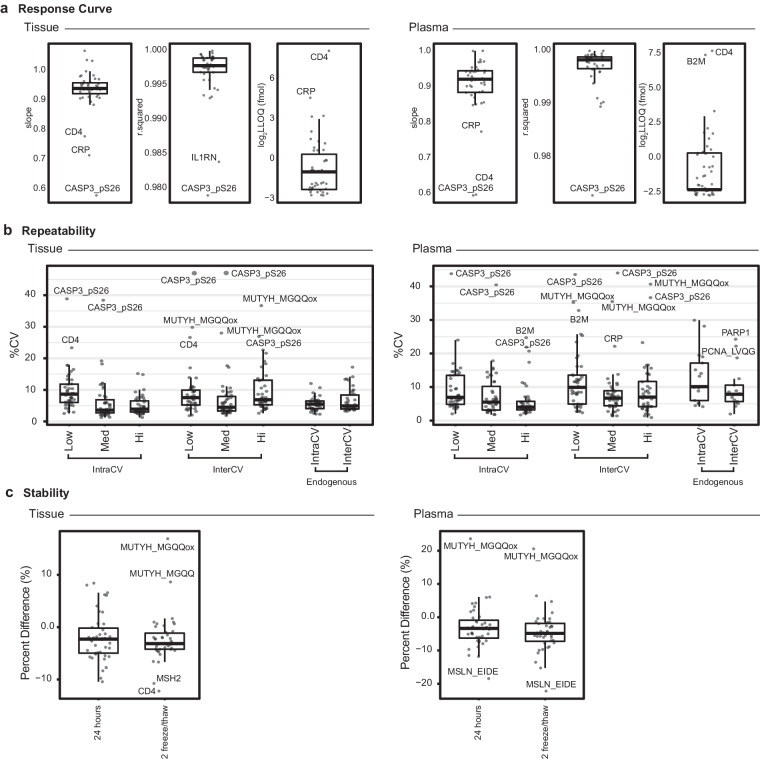
Characterization of performance figures of merit for the IO-3 panel in tissue and plasma matrices. (**a**) Metrics from response curves used to characterize the linearity and lower limits and quantification for the assay targets. Box plots show the slopes and correlation coefficients from linear regression of the response curves. LLOQs are reported for the lowest point on the response curve with CV < 20%. (**b**) Repeatability was determined using three samples (low, medium, high) measured in triplicate over five days. Box plots show the distribution of percent CVs for within-day (IntraCV), between-day (InterCV), and measurement of endogenous peptide > LLOQ in tissue and plasma. (**c**) Stability of the peptides was determined by comparing the peak area ratio of peptides stored at 24 hours at 4 °C or after 2 freeze/thaw cycles to the peak area ratio of peptides from a fresh aliquot of the same sample(s). For all box plots, the horizontal line shows the median, the box shows the inter quartiles, and the vertical line shows 5–95% of the data.

Figures of merit for linearity and limits of quantification were established using response curves applyinging the multiplexed IO-3 panel in two matrices: tissue and plasma (Data Record 3)^23^. Fig. 2a shows the distribution of slopes, correlation coefficient squared (R2), and lower limits of quantification (LLOQs) in tissue and plasma. LLOQs were determined by the lowest point on the curve with CV < 20%. The results of the response curves were very similar in tissue and plasma. Median dynamic range was ≥ 3.6 orders of magnitude in tissue and plasma matrices. Because the curves for most assays remained in the linear range at the highest point measured, the dynamic range determination is a minimum. The median LLOQs were 0.512 fmol in tissue (range 0.205-200 fmol) and 0.205 fmol in plasma (range 0.205-200 fmol). Three peptides (CRP_APLT, CD4_IDIV, and CASP3_pS26_IIHG) were consistently outliers in the curve results, showing lower dynamic range and relatively high LLOQs. This is likely the result of either low antibody activity or poor response of the peptide in the mass spectrometer. In addition, there was one peptide (B2M_VNHV) with notably different LLOQs in tissue (3.2 fmol) compared to plasma (200 fmol). This may indicate decreased performance of the antibody when used in plasma or suppression of the signal in the mass spectrometer from non-specifically bound plasma components.

### Repeatability

Repeatability of the multiplexed panel, defined as multiple measurements made by the same team of people and experimental setup over a short period of time, was determined by measuring three amounts of spiked peptides (20, 200, 2000 fmol; low, medium, high) and endogenous analyte in tissue and plasma matrices (Data Record 3)^23^. Fig. 2b shows the distribution of percent CVs for the peptides measured in tissue and plasma. The median intra-assay variability was 8.6%, 3.6%, and 3.9% (range 1.2–38%) for low, medium, and high tissue samples. Median inter-assay variability was 7.5%, 4.5%, and 6.9% (range 1.8–47%) for low, medium, and high tissue samples, respectively. For detection of endogenous analytes > LLOQ in tissue, the median intra-assay variability was 5.5% (range 2.2–12%) and median inter-assay variability for endogenous analytes was 4.9% (range 3.2–17%). The peptides CD4_IDIV and CASP3_pS26_IIHG failed validation in tissue matrix with multiple sample concentrations showing CV >20%. These peptides also exhibited poor performance in the response curve experiments. In addition, measurement of the oxidized methionine form of the peptide MGQQVLDNFFR (MUTYH_MGQQox) showed inter-assay variability >20% CV in each concentration of spiked samples. This could be due to poor stability of the methionine (see below). Note, the unoxidized form of the peptide exhibited acceptable repeatability performance (CV <20% in all samples).

In plasma, the median intra-assay variability was 6.9%, 5.5%, and 4.0% (range 1.7–44%) for low, medium, and high samples, respectively. Median inter-assay variability was 9.9%, 6.7%, and 7.0% (range 0.9–44%) for low, medium, and high tissue samples. The median intra-assay variability of endogenous plasma analytes > LLOQ was 10.1% (range 4.2–30%) and median inter-assay variability for endogenous analytes was 7.8% (range 1.9–24%). Similar to the tissue matrix results, CASP3_pS26_IIHG and MUTYH_MGQQox peptides failed validation in plasma matrix. We also found the peptide B2M_VNHV to have CV > 20% in the high-level intra-CV and low-level inter-CV experiments. There were also two peptides, PARP1_EELG and PCNA_LVQG, that had endogenous repeatability CV > 20%, likely due to low signals.

### Stability

Stability of the peptides was assessed in tissue and plasma under two conditions: (i) storing processed samples on the autosampler for 24 hours and (ii) subjecting samples to two freeze-thaw cycles prior to analysis (Data Record 3)^23^. Test samples were analyzed in process triplicate and results compared to aliquots of the same sample analyzed immediately. The percent difference is plotted in Fig. 2c. Overall, median differences were −2.3% and −3.1% for tissue and −3.4% and −4.8% in plasma. In tissue, the peptide MSH2_LYQG had differences of −10% under both conditions. The peptides CD4_IDIV and the oxidized MUTYH_MGQQox had differences > 10% after two freeze-thaw cycles, indicating these should be analyzed fresh. The same peptides were found to be less stable in plasma as well. Results show the peptides MS4A1_EEVV (differences of −11%) and MSLN_EIDE (differences of −18 to 22%) also required immediate analysis in plasma.

### Combination with other immuno-MRM assay panels

**Fig. 3:**
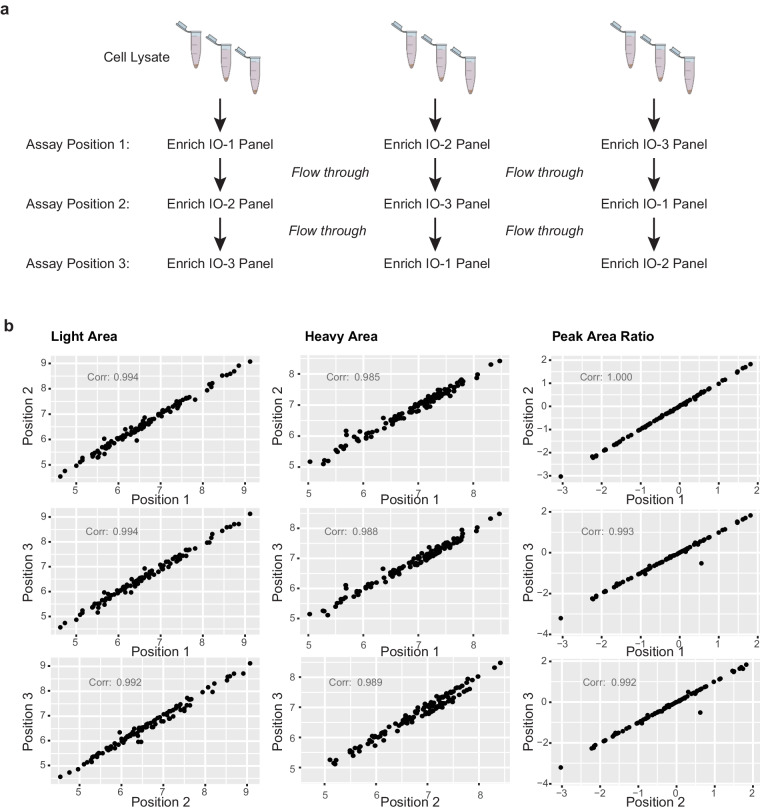
The IO-3 panel can be used in combination with other immuno-MRM panels. (**a**) Experimental design showing sequential enrichment of analytes in the IO-1, IO-2, and IO-3 panels from cell lysates. The flow-through from one enrichment is used to capture analytes from subsequent panels. The order of enrichment was varied such that analytes from the panels were tested in each configuration. (**b**) Correlation of light peak area, heavy peak area, and peak area ratio (light/heavy) for peptides from the IO-1, IO-2, and IO-3 panels measured > LLOQ after enrichment in positions 1–3 from the cell lysate.

The use of multiple distinct immuno-MRM assay panels can be used on a single specimen through taking the flow-through from the immunoaffinity enrichment step of one assay to perform enrichment of a separate panel of assays with different antibodies^14,17,46^. Application of assays in this manner allows for increasing the number of peptides that can be measured from a single sample, sparing precious biospecimens and reducing the cost and turnaround time of analysis. We validated the performance of the IO-3 panel in combination with previously described IO-1 and IO-2 panels, by using the flow-through of one assay for a subsequent enrichment (Data Record 4)^26^. We designed an experiment to enrich analytes from aliquots of a cell line lysate using each panel, IO-1 through IO-3, at each possible order of enrichment (Fig. 3a). For example, IO-3 analytes were enriched from a freshly digested sample (i.e., position 1), the flow-through of enrichment of IO-2 analytes (i.e., position 2), and the flow-through from enrichment of IO-1 and IO-2 analytes (i.e., position 3). Each test sample was analyzed in process triplicate, and we compared results from all analytes above LLOQ in the assays at each position of enrichment. Figure 3b shows the correlation of median light (endogenous) peak area, heavy (internal standard) peak area, and peak area ratio (light:heavy) for peptides > LLOQ at each enrichment position. Overall, there was excellent correlation (ranging 0.985 – 1.000), confirming that the IO-3 panel can be used sequentially with the other IO panels on a single sample to expand the number of analytes that can be measured.

### Utility of the assays in biological specimens

**Fig. 4:**
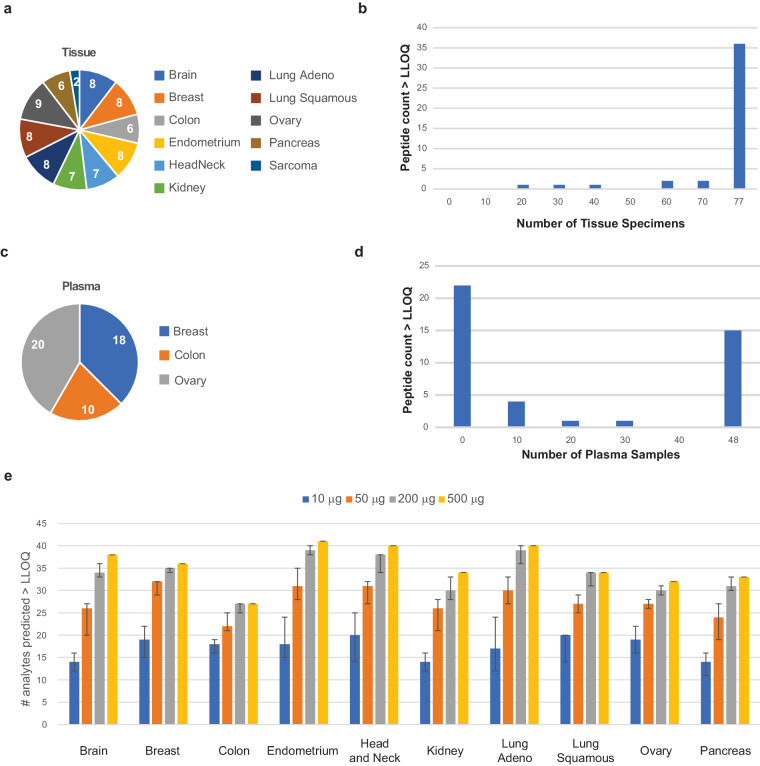
Utility of endogenous protein quantification in tissues and plasma. (**a**) A panel of frozen tissues was obtained for 77 tumors from 11 tumor types. The pie chart shows the number of each type. (**b**) Histogram of peptide detection, showing the number of peptides detected above LLOQ across the 77 frozen tumors. (**c**) A panel of plasma samples was obtained from 48 patients with breast, colorectal, or ovarian tumors, as indicated in the pie chart. (**d**) Histogram of peptide detection, showing the number of peptides detected above LLOQ across the 48 plasma samples. (**e**) Bar plot showing the number of analytes predicted to be above the LLOQ for decreasing amounts of input tissue lysate. Detection prediction was extrapolated from the signal-to-noise ratio measured in (**b**). Error bars show the 95% confidence interval.

We next sought to determine the number of analytes that can be detected at endogenous levels in clinical biospecimens (Data Record 5)^26^. This is important for evaluating the utility of the assay for measuring immunomodulatory proteins in biological specimens. To determine which proteins can be readily detected in cancer tissue biospecimens, we applied the IO-3 multiplexed panel to 77 frozen biopsy specimens collected from 11 different tumor types, including brain, breast, colorectal, endometrium, head and neck, kidney, lung (adenocarcinoma and squamous cell carcinoma), ovarian, pancreas, and soft tissue sarcoma (Fig. 4a). Figure 4b shows a histogram of peptide detection in the tissue biospecimens. Using 500 µg aliquots of the frozen tissue lysates, 36/43 peptides were detected in all 77 samples, and all 43 peptides were detected above LLOQ in at least 25% of the tissues. Three peptides (CTLA4_GIAS, CASP3_pS26_IIHG, MUTYH_MGQQox) were detected in less than half of the specimens. This is not surprising because CTLA4 is a low abundance protein, CASP3_pS26 failed validation, and we do not expect to find large amounts of oxidized methionine in the freshly prepared sample.

We also applied the assays to 48 plasma samples from patients with breast, colorectal, or ovarian cancer to evaluate the detection of endogenous proteins in circulation (Fig. 4c). 100 µL aliquots of each plasma sample were analyzed using the IO-3 assay. Overall, 21/43 peptides, corresponding to 18 proteins, were detected above LLOQ in the plasma samples (Fig. 4d). There were 22 peptides not detected in this set of plasma samples, indicating measurement of these targets in future plasma-based studies may not be feasible. These results show that although the immune-related proteins targeted in the IO-3 assay were selected for detection in the tumor microenvironment, a subset is also detectable in circulation.

Because the amount of clinical biospecimens available for analysis may be limited in many studies, we used the results from measurements in the 500 µg aliquots of the frozen tissue lysates to extrapolate the sample requirements for analyte detection in smaller quantities of input tissue lysate. Using the expression levels measured for each protein in the frozen tissue array, we estimated the minimum amount of tissue lysate needed to detect each analyte at endogenous levels. Figure 4e shows the number of peptides predicted to be detected above the LLOQ for tissue lysate inputs of 10, 50, 200, and 500 µg. There is some variation between tumor sites, but overall, the number of peptides predicted to be detected is between 51–74% with ten-fold less input. This indicates that most of the assays are amenable to a smaller amount of input. This estimation will help inform study design and sampling requests to optimize detection of endogenous analytes in future studies.

### Evaluation of the antibodies for use in other assay formats

In parallel to the development of the IO-3 immuno-MRM assay, we evaluated the anti-peptide antibodies for reactivity to the target proteins in traditional immunoassay approaches. Summary data tables are available in Data Record 6^23^, and additional images are available via the CPTAC Antibody Portal (antibodies.cancer.gov). Antibodies were first tested in traditional Western blotting using purified recombinant proteins (where available) and/or over-expressed lysates. Blots were considered positive if a prominent band at the expected molecular weight (given by the vendor for the recombinant proteins or based on the molecular weight reported in UniProt.org) was observed. For the IO-3 antibodies, 49% (19/39) of the antibodies tested were positive in Western blot against recombinant proteins, whereas 30% (12/40) were positive in Western blot against cell line or tissue lysates. Further testing was conducted using the Simple Wes platform and RPPA on over-expressed lysates. Overall, 13% (5/40) were positive against lysates in the Wes, 25% (10/40) were positive against lysates in RPPA, and 13% (1/8) were positive against lysates in immunofluorescence. These results were consistent with expectations based on previous work^47^. Antibodies deemed positive in the Western and RPPA experiments were further tested in immunohistochemistry (IHC) and protein immunoprecipitation (IP). For the antibodies tested, 53% (8/15) were positive in IHC. No antibodies tested (0/5) were found positive in protein immunoprecipitation.

## Usage Notes

The dataset described contains reagents, mass spectrometer parameters, and characterization data for a novel IO-3 multiplexed immuno-MRM assay panel developed to quantify 43 peptides corresponding to 39 proteins related to immuno-oncology. The assay uses targeted mass spectrometry, which is capable of multiplexing measurement of the peptides into a single run with high precision. Care was taken to select proteotypic peptides that best represent the target protein of interest by the avoidance of several common modifications; however, it is extremely challenging to anticipate all potential isoforms, variants, or modifications that may be present in the targeted peptide sequences. If present, these rare occurrences can affect the inferred protein concentration. When characterized, they can be added to the assay through inclusion of the appropriate peptide sequence(s). Additionally, the release of tryptic peptides following digestion may not be perfectly stoichiometric; however, the assay was shown to be highly reproducible (median %CV < 10) over multiple days/digestions. Thus, the assay shows accurate quantification of the reproducibly released tryptic peptides.

The assay panel has been characterized for application in tissue and plasma biospecimens but is generally amenable to other biofluids or preserved samples, including formalin fixed paraffin embedded (FFPE) tissue. In addition to the data records, assay characterization data and protocols are available at NCI’s CPTAC Assay Portal (assays.cancer.gov)^48^. The monoclonal antibodies and extensive characterization data are available through NCI’s CPTAC Antibody Portal (antibodies.cancer.gov).

The assay described expands on the development of multiplexed quantitative IO-1 and IO-2 assay panels, previously described, targeting immuno-modulatory proteins. Used together, the IO-1, IO-2, and IO-3 panels allow for analysis of 131 peptide analytes from a biospecimen with wide dynamic range, excellent precision, and high specificity. The assays may be used in correlative studies to support identification of new biomarkers for therapeutic efficacy or the prediction of adverse events. Furthermore, the quantitative proteomic readout can aid mechanistic studies aimed at tumor susceptibility and/or response to new therapies.

## Data Availability

No custom code was used in the dataset.
